# Nrf2 Is Required for Optimal Alveolar-Macrophage-Mediated Apoptotic Neutrophil Clearance after Oxidant Injury

**DOI:** 10.3390/antiox11020212

**Published:** 2022-01-22

**Authors:** Narsa M. Reddy, Chandra Mohan Tamatam, Ankireddy Aparna, Sekhar P. Reddy

**Affiliations:** 1 Department of Pediatrics, College of Medicine, The University of Illinois Chicago, Chicago, IL 60612, USA; NMReddy@northwestern.edu (N.M.R.); tamatam@uic.edu (C.M.T.); aparna1@uic.edu (A.A.); 2 Department of Pathology, The University of Illinois Chicago, Chicago, IL 60612, USA; 3 University of Illinois Chicago Cancer Center, The University of Illinois Chicago, Chicago, IL 60612, USA

**Keywords:** inflammation resolution, efferocytosis, oxidant stress, cell death, phosphatidylserine

## Abstract

Recognition and clearance of apoptotic cells by phagocytes (also known as efferocytosis), primarily mediated by macrophages, are essential to terminate lung inflammatory responses and promote tissue repair after injury. The Nrf2 transcription factor is crucial for cytoprotection and host defense. Previously, we showed sustained neutrophilic lung inflammation in Nrf2-deficient (*Nrf2*^−/−^) mice after hyperoxia-induced lung injury in vivo, but the mechanisms underlying this abnormal phenotype remain unclear. To examine whether Nrf2 regulates apoptotic neutrophil clearance, we used the alveolar macrophages (AMФs) and bone-marrow-derived macrophages (BMDMФs) of wild-type (WT) and *Nrf2*^−/−^ mice. We found that the efferocytic ability of AMФ was impaired in hyperoxia-exposed mice’s lungs, but the effect was more pronounced in *Nrf2*^−/−^ mice. Importantly, AMФ-mediated efferocytosis remained impaired in *Nrf2*^−/−^ mice recovering from injury but was restored to the basal state in the wild-type counterparts. Hyperoxia affected apoptotic neutrophil binding, not internalization, in both WT and *Nrf2*^−/−^ BMDMФs, but the effect was more significant in the latter cells. Augmenting Nrf2 activity restored hyperoxia attenuated efferocytosis in WT, but not in *Nrf2*^−/−^ macrophages. However, the loss of Nrf2 in neutrophils affected their uptake by WT macrophages. Collectively, these results demonstrate that Nrf2 is required for optimal macrophage-mediated efferocytosis and that activating Nrf2 may provide a physiological way to accelerate apoptotic cell clearance after oxidant injury.

## 1. Introduction

Acute lung injury (ALI) and acute respiratory distress syndrome (ARDS) are major clinical syndromes with significant mortality and morbidity rates [1]. The main pathological features of ALI and ARDS are loss of alveolar and endothelial barrier integrity and cell death, leading to lung edema and persistent neutrophilic inflammation [1]. While our understanding of the mechanisms underlying ALI and ARDS pathogenesis is evolving, poor clinical health outcomes, including mortality, persist in these patients [1]. Clearance of apoptotic cells in the injured tissues by phagocytes (known as efferocytosis), mainly by macrophages, is critical to terminate the local inflammatory response [2,3,4,5]. The imbalance between apoptotic cell death and efferocytosis can cause inflammation and organ dysfunction, leading to various inflammatory disorders, including ALI and ARDS [4,6,7]. Furthermore, inefficient removal of dying cells after injury prolongs and impairs inflammation resolution, impeding tissue repair [7,8].

Efferocytosis is a multistep, coordinated process mediated by multiple proteins and molecules. Dead or dying cells release signals (e.g., ATP, UTP, CX3CL1, and S1P) to initiate their recognition by resident phagocytes, largely macrophages [9]. Phosphatidylserine (PtdSer) exposure on the outer plasma membrane of the apoptotic cells serves as a critical recognition signal for their uptake by phagocytes [10]. Macrophages express multiple PtdSer receptors, such as Tim-1, Tim-4, BAI-1, Stabilin-1 and -2, CD36, SRA-1, αvβ3 integrins, and MerTK [11,12]. Apoptotic cell binding to the phagocyte triggers cytoskeletal remodeling, leading to the uptake and digestion of apoptotic cells [13]. Both resident and recruited macrophage subsets play critical roles in engulfing apoptotic cells in the lungs induced by both infectious and non-infectious injuries [3,14]. Crosstalk between macrophages and other cell types (e.g., T cells) [15] also plays a vital role in efferocytosis and inflammation resolution. However, the exact mechanisms underlying optimal or impaired efferocytosis after oxidant-induced lung injury remain poorly understood.

The nuclear factor (erythroid-derived 2)-like 2 (NFE2L2, also known as Nrf2) is a bZIP transcription factor crucial for cytoprotection and host defense functions [16]. Previously, we and others have reported that Nrf2 confers protection against prooxidant- and pathogen-induced lung injuries [16]. Furthermore, we found increased levels of persistent lung inflammation in Nrf2-deficient mice after hyperoxic lung injury [17] and increased susceptibility to subsequent infection after injury [18]. Because impaired efferocytosis contributes to persistent lung inflammation, we investigated whether Nrf2 regulates apoptotic neutrophil clearance and reduces lung inflammation after injury. In this paper, we report that Nrf2 is required for optimal alveolar macrophage (AMФ)-mediated apoptotic neutrophil clearance and that the boosting of endogenous Nrf2 activity mitigates the suppressive effects of hyperoxia on macrophage-mediated efferocytosis.

## 2. Materials and Methods

### 2.1. Mice and Hyperoxia Exposure

The wild-type (*WT* or *Nrf2*^+/+^) and Nrf2-deficient (*Nrf2*^−/−^) mice were exposed to hyperoxia or room air as previously described [17]. Mice cages were placed in a hyperoxia exposure chamber (Cat # A30274, BioSpherix, Ltd., Parish, NY, USA). The chamber bottom was lined with sufficient CO_2_ absorbent (Soda-sorb; W. R. Grace and Co., Lexington, MA, USA). Food and water were provided ad libitum. Sufficient humidified oxygen (Airgas, Brookfield, IL, USA) was delivered to the chamber continuously, and the oxygen concentration was adjusted to 100% and monitored with a Pro-Ox monitor (Model E702, BioSpherix, Ltd. Parish, USA). After 48 h of hyperoxia, a set of mice was allowed to recover in room air for 72 h. Animal protocols were approved by the Animal Care and Use Committee of the University of Illinois at Chicago.

### 2.2. Isolation and Induction of Apoptosis in Bone Marrow Neutrophils

Mature bone marrow neutrophils were isolated from mouse femurs and tibias [19]. Briefly, mice were euthanized, and bones were collected and flushed with HBSS. The marrow cells were centrifuged at 1600× *g* for 6 min, washed, resuspended in HBSS, and layered on top of a Percoll (P4937, Sigma-Aldrich, Saint Louis, MO, USA) density gradient (72%, 64%, and 52% with HBSS, 2 mL each). Cells were centrifuged at 1600× *g* for 30 min. Morphologically mature-appearing neutrophils formed a band at the interface of the 64% and 72% Percoll layers. This band was aspirated and mixed with HBSS, centrifuged at 1600× *g* for 6 min, washed twice with HBSS, and resuspended in RPMI-1640 medium without FBS and enumerated by a hemocytometer. Apoptosis in these neutrophils was induced by incubation at 43 °C for 60 min followed by 37 °C in 5% CO_2_ for 150 min [20]. These cells were used directly or stained with CellTracker Red (Thermo Fisher Scientific, Waltham, MA, USA) before incubating with macrophages.

### 2.3. Isolation and Culture of Bone-Marrow-Derived Macrophages (BMDMΦs)

Bone marrow cells were prepared and resuspended in ACK Lysing Buffer (Lonza, Rockville, MD, USA) to lyse the red blood cells. Cells were centrifuged and resuspended in RPMI-1640 supplemented with 10% FBS, 1% penicillin and streptomycin, and 10% conditioned medium collected from L929 cultures. Cells were grown for 5–7 days with medium replenishing on alternate days.

### 2.4. Ex Vivo Efferocytosis Assays

*Nrf2*^+/+^ and *Nrf2*^−/−^ mice were exposed to room air or 48 h hyperoxia. Some mice exposed to hyperoxia were allowed to recover at room air for 72 h. The mice were euthanized, and the bronchoalveolar lavage (BAL) was collected, centrifuged at 400× *g* for 5 min, and washed twice with PBS. BAL cells (5 × 10^4^) were incubated in RPMI-1640 complete medium on cover glasses for 30 min for macrophage adherence. Non-adherent cells were washed away with PBS. The adherent macrophages were incubated with 1 × 10^6^ apoptotic neutrophils. After one hour of incubation, unbound or non-internalized cells were washed away with PBS. The AMΦs with bound and internalized neutrophils on cover glasses were stained with a Diff-Quick Staining Kit and fixed in 4% paraformaldehyde. Cells were imaged, and the number of apoptotic neutrophils either bound or internalized by macrophages in different fields were quantified and are expressed as percent efferocytosis.

### 2.5. In Vitro Efferocytosis Assays

BMDMΦs were exposed to 95% O_2_ + 5% CO_2_ for 24 h in a modular exposure chamber. After hyperoxia exposure, macrophages were stained with carboxyfluorescein succinimidyl ester (CSFE), following the manufacturer’s instructions (Thermo Fisher Scientific, Waltham, MA, USA). CSFE-stained macrophages on cover glasses were incubated with apoptotic neutrophils labeled with CellTracker Red (20 µM) for 1 h. Finally, the unbound cells were washed with PBS three times and fixed in 4% paraformaldehyde. Efferocytosis was determined as described above. Additionally, BMDMΦs were incubated with carboxylate-modified polystyrene fluorescent (green) latex beads (L4655, Sigma-Aldrich, Saint Louis, MO, USA). For Nrf2 activation studies, BMDMΦs were treated with CDDO-Im (1-[2-cyano-3-,12-dioxooleana-1,9(11)-dien-28-oyl] imidazole) (20 nM) or DMSO (vehicle) during hyperoxia, and efferocytosis assays were performed with apoptotic neutrophils labelled with CellTracker Red.

### 2.6. Binding and Internalization Assays

BMDMΦs were exposed to hyperoxia or room air for 24 h, stained with CellTracker Red, and then incubated with cytochalasin D (15 μM) (Sigma-Aldrich, Saint Louis, MO, USA) for 20 min [21]. DMSO was used as the vehicle control. After washing the plate twice with PBS, BMDMΦs were incubated with CSFE-stained apoptotic neutrophils for 30 min. Finally, the unbound cells were washed with PBS three times and fixed in 4% paraformaldehyde. The images were captured, and efferocytosis was determined as described above.

### 2.7. Statistical Analysis

A two-way ANOVA analysis with Tukey’s multiple comparisons test with Prism 9 (GraphPad, San Diego, CA, USA) was used to calculate the significance between *WT* and *Nrf2*^−/−^ genotypes and treatment conditions. Error bars are shown with the SD. The symbols representing *p* values are: *, room air vs. hyperoxia; †, *WT* vs. *Nrf2*^−/−^ genotypes; §, DMSO vs. cytochalasin D or DMSO vs. CDDO.

## 3. Results

### 3.1. Nrf2 Deficiency Worsens Alveolar-Macrophage-Mediated Efferocytosis after Injury

**Figure 1:**
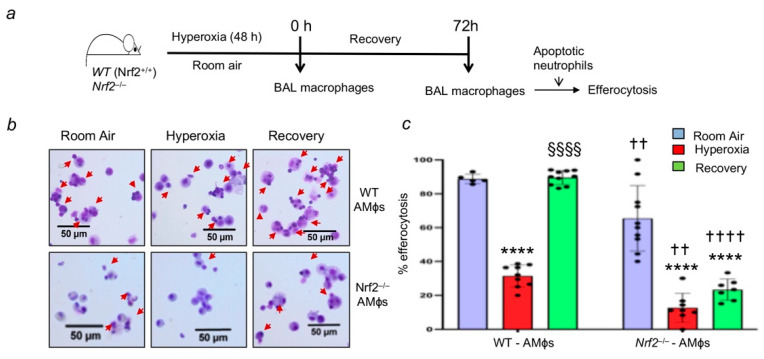
Hyperoxia impairs alveolar macrophage-mediated efferocytosis, and Nrf2-deficiency worsens it in vivo. *Nrf2*^+/+^ (WT) and *Nrf2*^−/−^ mice (*n* = 3 per group) were exposed to room air or hyperoxia for 48 h, and a set of hyperoxia-exposed mice were allowed to recover at room air for 72 as outlined in schema. (**a**) BAL from these mice was obtained, and macrophages were incubated on cover glasses with apoptotic neutrophils for 1 h. Macrophages were washed to remove unbound/non-internalized apoptotic cells and stained with Diff Quick stain. Images were captured to quantify apoptotic neutrophil binding, and internalization (indicated by red arrows). (**b**) Representative images of macrophages with neutrophils for each experimental condition are shown. (**c**) The number of apoptotic neutrophils either bound or internalized by macrophages from ~5–9 fields were quantified and expressed as % efferocytosis. Versus room air of respective genotypes, † *Nrf2*^−/−^ versus WT counterparts, § hyperoxia vs. recovery; †† *p* < 0.01; ****/§§§§/†††† *p* < 0.0001. Purple, room air group; Red, hyperoxia group; Green, hyperoxia and recovery.

Nrf2 deficiency causes persistent lung neutrophilic inflammation after hyperoxic lung injury [17]. Thus, we examined the role of Nrf2 in mediating efferocytosis. To rule out the effects of lung resident and infiltrated cells on efferocytosis in vivo, we performed studies ex vivo with AMФs isolated from the room-air-exposed and hyperoxia-exposed mice. We labeled apoptotic neutrophils as outlined in the schema in Figure 1a. The efferocytic ability of AMФs obtained from *Nrf2*^+/+^ and *Nrf2*^−/−^ mice exposed to hyperoxia was significantly lower than that of their room air counterparts (Figure 1b,c). However, the inhibition of efferocytosis was more pronounced in *Nrf2*^−/−^ AMФs s compared to that of *Nrf2*^+/+^ AMФs (Figure 1b,c). Furthermore, the efferocytic ability was restored to the basal state in AMФs of the wild-type mice recovered from hyperoxic injury. In contrast, AMФ-mediated efferocytosis remain impaired in *Nrf2*^−/−^ mice post-injury.

### 3.2. Oxidant Stress Impairs Macrophage-Mediated Efferocytosis and Nrf2 Deficiency Worsens It

**Figure 2:**
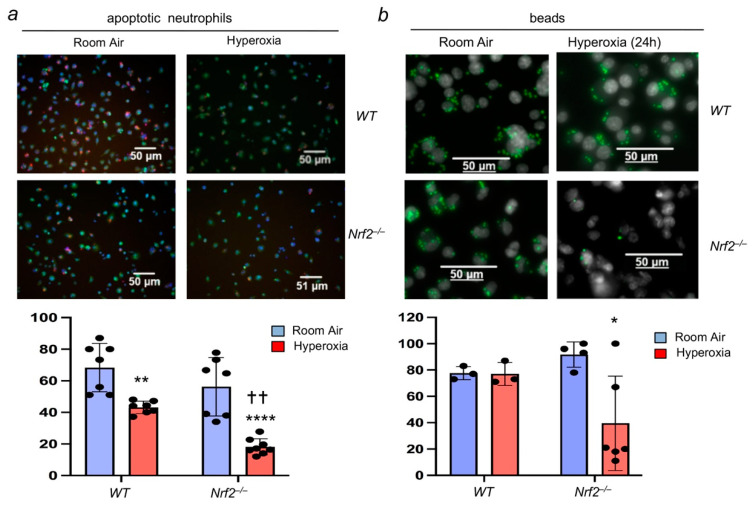
Nrf2 is required for optimal macrophage-mediated efferocytosis. BMDMФs from *Nrf2*^+/+^ (WT) and *Nrf2*^−/−^ mice were cultured and exposed to hyperoxia for 24 h. (**a**) Macrophages (green) were then incubated with labeled apoptotic neutrophils (red) for 1 h, washed, and captured images. (**b**) Macrophages were incubated with green fluorescent beads. Representative images of macrophages with neutrophils or beads for each genotype and experimental condition are shown. Values represent from at least three independent samples. * Room air versus hyperoxia of respective genotypes, †, *Nrf2*^−/−^ versus WT counterparts. * *p* < 0.05; **/†† *p* < 0.01; **** *p* < 0.0001. Blue, room air group; Red, hyperoxia group.

To directly examine the role of Nrf2 in mediating efferocytosis, we performed studies with BMDMФs isolated from *Nrf2*^−/−^ and *Nrf2*^+/+^ mice. BMDMФs were exposed to room air or hyperoxia for 24 h and incubated with the apoptotic neutrophils. Efferocytosis mediated by macrophages exposed to hyperoxia in vitro was significantly reduced in both *Nrf2*^−/−^ and *Nrf2*^+/+^ cell types (Figure 2), and these results are consistent with the in vivo data (Figure 1). However, the magnitude of inhibition was more pronounced in *Nrf2*^−/−^ macrophages exposed to hyperoxia (Figure 2a). Additionally, we performed efferocytosis assays using fluorescent beads to verify the results (Figure 2b). Similar to the apoptotic neutrophils, studies with beads revealed impaired efferocytosis in *Nrf2*^−/−^ cells exposed to hyperoxia. Taken together, these results demonstrate that hyperoxia dampens macrophage-mediated efferocytosis and Nrf2 deficiency further impairs it. Notably, unlike apoptotic neutrophils, no differences between the bead bindings were noted in room-air-exposed *Nrf2*^−/−^ and *Nrf2*^+/+^ BMDMФs and *Nrf2*^+/+^ BMDMФs exposed to hyperoxia. This discrepancy could be attributed to the size and or cell surface differences between apoptotic neutrophils and inert beads.

### 3.3. Nrf2 Deficiency in Apoptotic Neutrophils Does Not Affect Their Efferocytosis by Macrophages

**Figure 3:**
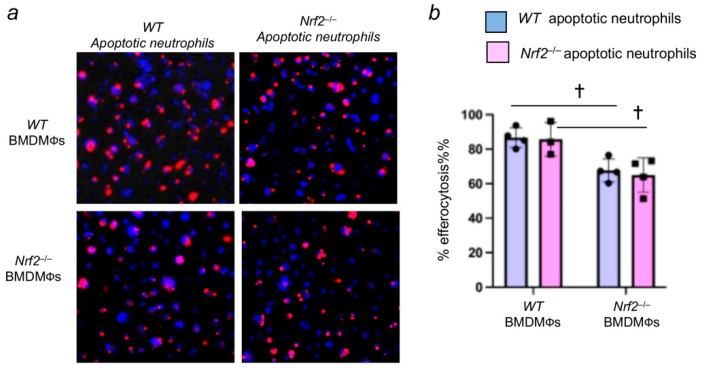
Nrf2-deficiency in neutrophils does not affect their engulfment by macrophages. Neutrophils from the bone marrow of wild-type (WT) and *Nrf2*^−/−^ mice were isolated, labelled, and subjected to apoptosis as detailed in methods. Apoptotic neutrophils (red) were incubated with WT BMDMФs and *Nrf2*^−/−^ BMDMФs for 1 h, and efferocytosis was quantified. (**a**) Representative images of macrophages with apoptotic neutrophils (red). The blue color represents DAPI. (**b**) quantification of efferocytosis. Values are from 3–4 fields of two independent samples. † *p* < 0.05; *Nrf2*^−/−^ versus WT counterparts. Blue, room air group; pink hyperoxia group.

We next evaluated whether Nrf2 deficiency in apoptotic neutrophils affects their recognition by macrophages, thereby contributing to persistent neutrophilic lung inflammation in *Nrf2*^−/−^ mice [17,18]. To assess this aspect, apoptotic neutrophils from the bone marrow of *Nrf2*^+/+^ and *Nrf2*^−/−^ mice were isolated, stained with CellTracker Red, and incubated with the BMDMФs of *Nrf2*^+/+^ or *Nrf2*^−/−^ mice. The efferocytosis was enumerated as detailed above. We found that *Nrf2*^+/+^ or *Nrf2*^−/−^ apoptotic neutrophils were bound and internalized by wild-type *(Nrf2^+/+^*) macrophages in equal proportion (Figure 3a). However, as anticipated, efferocytosis of *Nrf2*^+/+^ or *Nrf2*^−/−^ apoptotic neutrophils by *Nrf2*^−/−^ macrophages was significantly lower than that of wild-type macrophages (Figure 3b). These results suggest that the lack of Nrf2 in apoptotic neutrophils does not affect their engulfment ability by macrophages.

### 3.4. Nrf2 Regulates Apoptotic Neutrophil Binding

**Figure 4:**
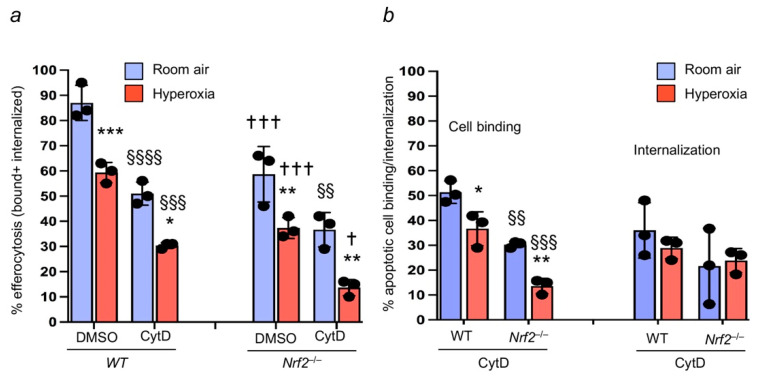
Nrf2 regulates apoptotic neutrophil binding to the macrophages. *Nrf2*^+/+^ (WT) and *Nrf2*^−/−^ BMDMФs were exposed to room air or hyperoxia and then incubated with cytochalasin D (CtyD) before adding the labeled apoptotic neutrophils. Efferocytosis was quantified. (**a**) Both bound/attached and internalized apoptotic cells in the presence and absence of CytD were enumerated, and data are represented % efferocytosis. * hyperoxia versus room air of respective genotypes, †, *Nrf2*^−/−^ versus WT counterparts; § DMSO versus CtyD of respective genotypes. */† *p* < 0.05; **/§§ *p* < 0.01; ***/†††/§§§ *p* < 0.001; §§§§ *p* < 0.0001. Blue, room air group; Red, hyperoxia group. (**b**) Both bound and internalized apoptotic cells were enumerated by comparing values of respective CytD (bound) and DMSO-treated (bound and internalized) samples, * Hyperoxia versus room air of respective genotypes, and §, *Nrf2*^−/−^ versus WT versus counterparts. Values are from three independent samples. * *p* < 0.05; **/§§ *p* < 0.01; §§§ *p* < 0.001. Blue, room air group; Red, hyperoxia group.

We next determined whether Nrf2 regulates apoptotic neutrophil internalization in addition to apoptotic binding to macrophages. Cytochalasin D does not interfere with apoptotic cell binding to the macrophages but prevents apoptotic cell internalization by inhibiting actin polymerization [21]. Therefore, *Nrf2*^+/+^ and *Nrf2*^−/−^ BMDMФs were exposed to room air or hyperoxia and immediately treated with cytochalasin D before incubating with the apoptotic neutrophils. As expected, hyperoxia dampened the efferocytosis in DMSO-treated BMDMФs of both genotypes, but the effect was more prominent in Nrf2-deficient cells than in the *Nrf2*^+/+^ counterparts (Figure 4a). Treatment of cells with cytochalasin further attenuated efferocytosis mediated by *Nrf2*^+/+^ and *Nrf2*^−/−^ macrophages (Figure 4a). However, efferocytosis was further inhibited in cytochalasin-treated *Nrf2*^−/−^ cells compared to the *Nrf2*^+/+^ counterparts (bars 2 and 4 versus bars 6 and 8, Figure 4a). In addition, we found significantly decreased levels of apoptotic cell binding to the Nrf2-deficient macrophages compared to the WT counterparts (Figure 4b). The apoptotic cell internalization rate was modestly reduced in *Nrf2*^−/−^ cells (29% versus 24%, *WT* versus *Nrf2*^+/+^).

### 3.5. Increasing Endogenous Nrf2 Activity Stimulates Macrophage-Mediated Efferocytosis

**Figure 5:**
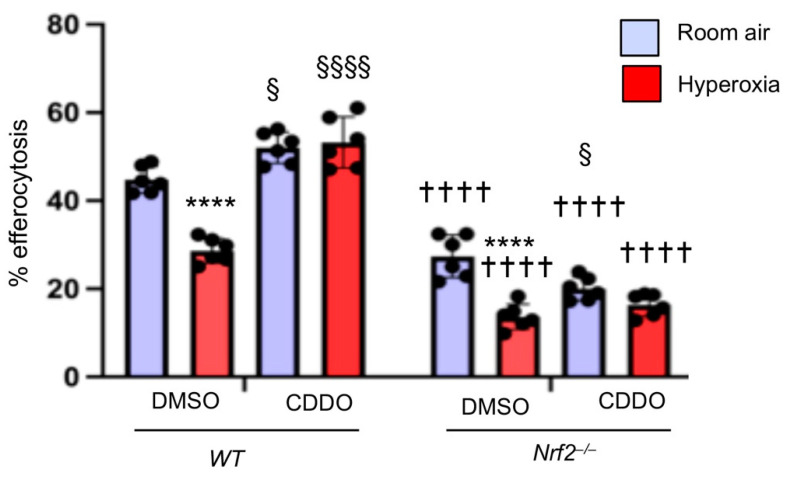
Nrf2 activation augments efferocytosis in hyperoxia exposed BMDMΦs. *Nrf2*^+/+^ and *Nrf2*^−/−^ BMDMΦs were pretreated with CDDO-Im (20 nM) for 6 h and exposed to hyperoxia for 24 h along with CDDO-Im. After hyperoxia, BMDMΦs were incubated with CellTracker Red stained apoptotic neutrophils for 1 hour. Images were captured, and % efferocytosis was enumerated. Values are at least from three independent samples. * Hyperoxia versus room air of respective genotypes, † versus WT counterparts, and § DMSO versus CDDO of respective genotypes. § *p* < 0.05; ****/††††/§§§§ *p* < 0.0001. Blue, room air group; Red, hyperoxia group.

Above studies demonstrated that hyperoxia impairs macrophage-mediated efferocytosis, and Nrf2 deficiency worsens it. Thus, we examined whether increasing the endogenous Nrf2 activity in macrophages would mitigate hyperoxia-induced dampening effects on efferocytosis. To evaluate this aspect, BMDMΦs were treated with vehicle or CDDO (Nrf2 activator) [22] for 6 h and then exposed to hyperoxia or room air for 24 h. BMDMΦs were incubated with apoptotic neutrophils, and efferocytosis was enumerated. As shown in Figure 5, hyperoxia suppressed the efferocytic ability of BMDMΦs by 30% compared to that of the room-air-exposed counterparts (left panel). However, CDDO mitigated the suppressive effect of hyperoxia on macrophage-mediated efferocytosis (right panel). We also treated *Nrf2*^−/−^ BMDMΦs with CDDO to verify whether this compound confers protection through Nrf2. In contrast to its protective effects in WT cells, CDDO did not prevent the suppressive effects of hyperoxia on efferocytosis in Nrf2-deficient BMDMΦs.

## 4. Discussion

The present study revealed the essential role of Nrf2 in regulating macrophage-mediated apoptotic neutrophil clearance, which is crucial for resolving lung inflammation after oxidant injury. Furthermore, we found that oxidant stress (hyperoxia) dampens macrophage-mediated efferocytosis, and Nrf2 deficiency worsens it. Increasing Nrf2 activity, however, mitigated the detrimental effects of hyperoxia on macrophage-mediated efferocytosis. Recognition and clearance of apoptotic lung resident cells and infiltrated neutrophils by macrophages is crucial to effectively terminating the local inflammatory response and promoting tissue repair after injury [2,7]. Defective efferocytosis may accumulate apoptotic cells, leading to secondary necrosis, chronic inflammation, and tissue damage. Thus, dysfunctional macrophage Nrf2-mediated efferocytosis may be attributed to a mechanism underlying persistent lung inflammation in Nrf2-deficient mice after pro-oxidant-induced lung injury [17,23,24,25].

Several studies have shown that hyperoxia compromises innate immunity following infection by impairing macrophage-mediated phagocytosis [26,27,28,29]. It was demonstrated that endotoxin- and cigarette-smoke-induced cellular stress both alter the expression levels of phagocytic receptors in macrophages [30] or promote their shedding from macrophages [31], thereby affecting efferocytosis. Antioxidant supplementation improved macrophage functions and mitigated hyperoxia-induced lung injury [32]. In addition, resolvins [33,34], statins, and pioglitazone have been shown to enhance macrophage-mediated apoptotic cell clearance [35,36,37]. The present study revealed that hyperoxia dampens macrophage-mediated efferocytosis, and Nrf2 deficiency worsens it. Previously, we found elevated levels of oxidative stress in the alveolar macrophages of wild-type and Nrf2-deficient mice exposed to sublethal-hyperoxia [18]. Thus, increased cellular stress induced by hyperoxia could impair macrophage-mediated efferocytosis, both in vivo and in vitro. We found that treatment of macrophages with CDDO, a potent activator of Nrf2-dependent antioxidant gene expression [22], mitigates the hyperoxia-induced suppressive effects on efferocytosis (Figure 5). Previously, we and others have shown that CDDO via the Nrf2 and ARE pathways confers protection from lung injury and inflammation [38,39,40]. Further studies are warranted on how hyperoxia attenuates efferocytosis and whether activating Nrf2 after oxidant injury enhances macrophage-mediated efferocytosis and inflammation resolution.

Our studies show that apoptotic neutrophils derived from *Nrf2*^−/−^ mice are equally recognized by wild-type macrophages, suggesting that the loss of Nrf2 in apoptotic cells does not impair their ability to be engulfed by the macrophages. The exposure of PtdSer on the outer plasma membrane of the apoptotic cells is recognized by several PtdSer receptors [10,11,12]. These receptors are expressed at different levels by both lung resident macrophages and recruited macrophages [41]. Previously, we and others have shown that Nrf2 is required for scavenger receptor MARCO induction by hyperoxia and cigarette smoke in lung macrophages [18,42]. 15-Deoxy-Δ12,14-prostaglandin J2 (15d-PGJ2) promoted efferocytosis through the induction of CD36 in peritoneal macrophages via Nrf2 [43]. Likewise, *Hmox1*, the Nrf2 target gene, plays a crucial role in mediating the engulfment of eryptotic red blood cells and subsequent Rac1 activity [44]. Taurine (amino sulfonic acid) released from the apoptotic neutrophils activates Nrf2 and stimulates Hmox1 expression, promoting macrophage-mediated efferocytosis with increased PtdSer expression [45]. However, cigarette smoke downregulates the efferocytotic receptor (e.g., milkfat globule-EGF factor 8) expression in macrophages [46]. Oxidative stress alters the extracellular environment and functions of membrane receptors and cytoskeletal actin remodeling, thereby affecting host defense functions mediated by macrophages, including efferocytosis [47,48,49,50]. Lung macrophages express multiple PtdSer receptors [41] that bind directly to the PtdSer or indirectly via various bridging molecules [2,11,12]. In silico analysis revealed Nrf2 binding sites (AREs) in the regulatory elements of some of the PtdSer receptors. Therefore, further studies are warranted to define whether Nrf2 deficiency leads to an altered state of selective efferocytotic machinery or if it affects the extracellular environment of the macrophages, resulting in reduced apoptotic neutrophil binding to the macrophages after oxidant injury. 

The present study has a few limitations. We performed efferocytosis studies ex vivo with macrophages obtained from the BAL of Nrf2-sufficient and Nrf2-deficient mice exposed to room air and hyperoxia. However, the cellular environment in the injured lung is likely different, and this could affect macrophage-mediated efferocytosis in vivo. In addition, efferocytotic processes, such as apoptotic cell binding, internalization, and digestion, occur rapidly. Thus, determining the nature of efferocytotic processes regulated by Nrf2 in vivo warrants additional tools, such as intravital and confocal imaging techniques. Another limitation is that macrophages obtained from the BAL of an injured lung likely comprise resident and recruited macrophages. Our studies with lung and monocyte-derived macrophages (BMDMФs) revealed that Nrf2 signaling in both cell types is required for optimal efferocytosis following exposure to oxidant stress. Whether Nrf2 signaling in lung resident (alveolar and interstitial) macrophages, infiltrated macrophages, or both is essential for mediating efferocytosis after infectious and non-infectious lung injuries remains unclear and warrants further study.

## 5. Conclusions

The current study revealed the crucial role of Nrf2 in mediating apoptotic neutrophil clearance, an essential step in the termination of lung inflammation after injury. Our findings suggest that increasing Nrf2 activation is sufficient to mitigate the detrimental effects of oxidant stress on macrophage-mediated efferocytosis. Whether activating Nrf2 after lung injury will accelerate the efferocytosis and reduce lung inflammation in vivo warrants further studies before exploring this strategy in the clinical setting.

## Data Availability

Data are contained within the article.
